# Swallowing Lithium Dendrites in All‐Solid‐State Battery by Lithiation with Silicon Nanoparticles

**DOI:** 10.1002/advs.202103786

**Published:** 2021-11-19

**Authors:** Jianming Tao, Daoyi Wang, Yanmin Yang, Jiaxin Li, Zhigao Huang, Sanjay Mathur, Zhensheng Hong, Yingbin Lin

**Affiliations:** ^1^ Fujian Provincial Solar Energy Conversion and Energy Storage Engineering Technology Research Center College of Physics and Energy Fujian Normal University Fuzhou 350117 China; ^2^ Fujian Provincial Key Laboratory of Quantum Manipulation and New Energy Materials Fuzhou 350117 China; ^3^ Fujian Provincial Collaborative Innovation Center for Advanced High‐Field Superconducting Materials and Engineering Fuzhou 350117 China; ^4^ Institute of Inorganic Chemistry University of Cologne Greinstr.6 Cologne 50939 Germany

**Keywords:** solid state batteries, solid electrolytes, lithium dendrites, Si nanoparticles

## Abstract

Eliminating the uncontrolled growth of Li dendrite inside solid electrolytes is a critical tactic for the performance improvement of all‐solid‐state Li batteries (ASSLBs). Herein, a strategy to swallow and anchor Li dendrites by filling Si nanoparticles into the solid electrolytes by the lithiation effect with Li dendrites is proposed. It is found that Si nanoparticles can lithiate with the adjacent Li dendrites which have a strong electron transport ability. Such effect can inhibit the formation of Li dendrites at the interface of Li anode, and also swallow the tip Li inside the solid electrolytes, and thus inhibiting its longitudinal growth and avoiding the solid electrolyte puncturing. As a proof of concept, a novel sandwich‐structure solid electrolyte of Li_6.7_La_3_Zr_2_Al_0.1_O_12_ (LLZA)‐PEO/Si‐PEO electrolyte/ (LLZA)‐PEO with asymmetrical structure is first constructed and demonstrated stable Li plating/stripping over 1800 h and remarkably improved cycling stability in Li/LiFePO_4_ cells with a reversible capacity of 111.9 mAh g^−1^ at 1 C after 150 cycles. The proof of lithiation of Si‐PEO electrolyte in the interlayer is also verified. Furthermore, the pouch cell thus prepared exhibits comparable cyclic stability and is allowable for folding and cutting, suggesting its promising application in ASSLBs by this simple and efficient strategy.

## Graphical abstract

Regulating the local growth of lithium dendrites inside solid electrolytes is essential for the application of all‐solid‐state Li metal batteries. In this work, a strategy to swallow and anchor Li dendrites by filling Si nanoparticles into the solid electrolytes by the lithiation effect with Li dendrites is proposed.

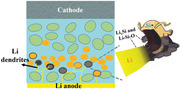

## 1 Introduction

Developing high‐energy density and safety batteries is highly desirable for the growing demand for energy consumption in large‐scale energy‐storage systems. All‐solid‐state Li batteries (ASSLBs) have been extensively investigated as the most promising candidates of the “beyond Li‐ion batteries” due to their high energy density, high safety and superior cycle stability.^[^ ^1^, ^2^, ^3^ ^]^ However, the ineluctable growth of Li dendrite caused by various indefinite physical and chemical problems makes ASSLBs difficult to achieve the desired long‐cycling life and high safety nowadays.^[^ ^4^, ^5^ ^]^ Typical solid electrolytes (SEs), such as garnet Li_7_La_3_Zr_2_O_12_ (LLZO) and sulfide electrolytes, have a high ionic conductivity and Li^+^ transfer numbers, but recent reports suggested that Li dendrites can still penetrate into the electrolyte.^[^ ^6^, ^7^, ^8^ ^]^ The high interfacial impedance, hostile interface reaction, grain boundary defects and the non‐negligible electronic conductivity, lead to the growth of Li dendrite, and cause the partial electrical short‐circuit in SEs.^[^ ^9^ ^]^ Although the polymer‐inorganic composite SEs has tamper force with mercy, it still cannot completely inhibit the growth of Li dendrite due to its adverse attributes including the low oxidation potential and poor ionic conductivity.^[^ ^10^, ^11^ ^]^

Li filament or dendrite formation in ASSLBs is generally rooted in the uneven residual stress, nonuniform heat distribution, heterogeneous current field and incongruent ion diffusion in SE/Li interface or inside SEs.^[^ ^12^, ^13^, ^14^, ^15^ ^]^ A. Gupta et al.,^[^ ^16^ ^]^ for example, observed directly the Li dendrites at 1 mA cm^−2^ at 80 °C in Li/PEO‐LiTFSI/Li cells by the operando optical observation, revealed that dendrites still exist at high temperatures despite the rapid Li diffusion. Tremendous strategies have been adopted to alleviate this issue, in short, to make SEs have high modulus,^[^ ^17^ ^]^ high ion diffusion,^[^ ^18^ ^]^ high steady interface,^[^ ^19^ ^]^ low enough interfacial resistance,^[^ ^20^, ^21^ ^]^ and keep equilibrium of the interfacial ion‐electron distribution with Li, and low enough but uniform interfacial stress.^[^ ^22^ ^]^ Madsen et al.^[^ ^23^ ^]^ reported a solid‐state molecular ionic composite electrolyte with high strength of 200 MPa, outstanding Li^+^ conductivity (1 mS cm^−1^ at 25 °C), which exhibits low interfacial resistance and overpotentials to maintain dendritic‐free running. Wang's group^[^ ^24^ ^]^ coated a Li_3_N‐LiF layer with high ionic conductive, high modulus and high interface energy on Li_3_PS_4_ to suppress the Li penetration into the SEs, and achieve a record‐high critical current of >6 mA cm^−2^. Nevertheless, it is substantially difficult to completely avoid the formation of Li dendrite in any SEs with the deepened knowledge about its nature.^[^ ^25^, ^26^ ^]^ In fact, the number of dendrites that infiltrate SEs to damage or pierce SEs seriously is not much but their harm is fatal.^[^ ^27^ ^]^ Eliminating the extension of these deadliest dendrites is undoubtedly more practical than the dendrite‐free model in application, and it is also very effective in improving the performance of ASSLBs.^[^ ^27^, ^28^ ^]^ Recently, Li et al.^[^ ^29^ ^]^ proposed a Li_5.5_PS_4.5_Cl_1.5_ (LPSCl)/Li_10_GeP_2_S_12_ (LGPS)/LPSCl multilayer electrolyte, which prevents any Li dendrite growth by anchoring Li dendrite and dynamically repairing cracks through the decomposition of the unstable LGPS interlayer. This strategy of anchoring Li dendrite inside SEs has proved to be promising, but is currently limited to a few materials and cell systems. Therefore, it is highly desirable to develop a general and simple strategy to anchor dendrites in the most SEs for unleashing the performance of ASSLBs.

Herein, motivated by the above concerns, we added Si nanoparticles into the polyoxyethylene (PEO)‐based solid electrolyte, and found its unusual properties of swallowing and anchoring Li dendrites by the lithiation effect. In addition, Si‐PEO based solid electrolytes display improved Li^+^ transport performance and can inhibit the PEO oxidation at high voltage. As a proof of concept, we report a novel sandwich‐structure electrolyte of Li_6.7_La_3_Zr_2_Al_0.1_O_12_ (LLZA)‐PEO hybrid solid electrolyte (HSE)/Si‐PEO electrolyte/HSE to improve the performance of ASSLBs. Profit from the outstanding Li^+^ diffusion ability of LLZA, and the Li dendrites swallowing effect by the Si nanoparticles at the interlayer, this sandwich electrolyte guarantees a steady Li plating/stripping over 1800 h in Li/Li symmetrical cells and demonstrates a specific capacity of 111.9 mAh g^−1^ at 1 C after 150 cycles in Li/LiFePO_4_ cells. Furthermore, the pouch cell fabricated by this electrolytes exhibits good cycling stability and superior flexibility.

## 2 Results and Discussion

**Figure 1:**
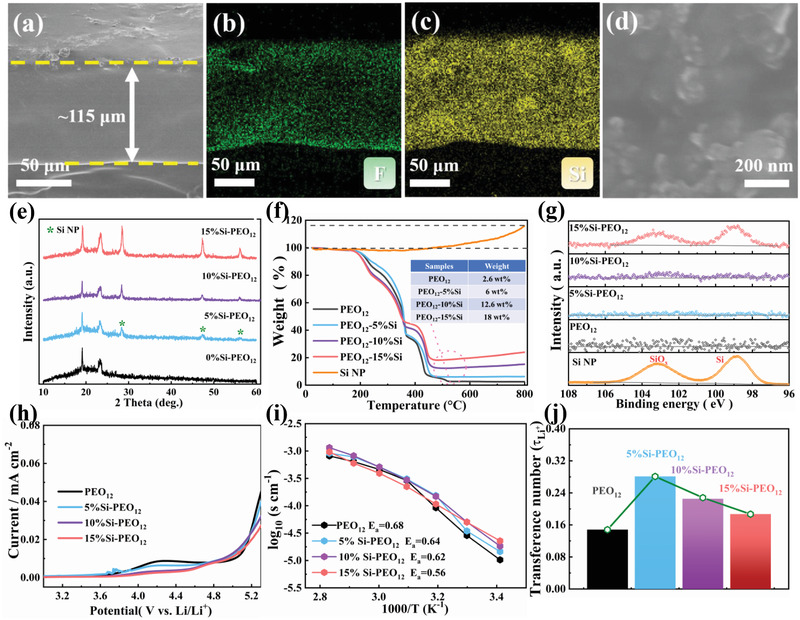
a) Cross‐sectional SEM image of 15%Si‐PEO_12_. b,c) The EDS elemental mapping images of F and Si for 15%Si‐PEO_12_. d) SEM image of 15%Si‐PEO_12_. e) XRD patterns of all typical electrolytes. f) TGA curves of the electrolytes and the corresponding calculated results illustrated in the interpolation table. g) The Si 2p spectrum of all typical electrolytes. h) LSV curves of all typical electrolytes at a scanning rate of 1 mV s^−1^ at 60 °C. i) Arrhenius plots of all typical electrolytes for the calculation of the activation energy. j) Li^+^ transfer number chart of all typical electrolytes.

First, Si‐PEO based solid films with different Si contents were fabricated to investigate their electrochemical properties upon Li^+^ transport and deposition. As displayed in Figure S1, Supporting Information, the Si nanoparticles (Si NPs) with a diameter of 50–100 nm were collected as an additive to penetrate into PEO‐based solid electrolyte. **Figure** **1**a and Figure S2, Supporting Information, further indicate that the thickness of all typical electrolytes is within the range of 110–130 µm. With increasing the contents of Si NPs, the roughness of electrolytes is slightly increased since polymer still occupies the main body. The EDS elemental mapping images (Figure 1b,c) and the SEM image of Si NPs (Figure 1d) indicate that Si is uniformly distributed in PEO‐based solid electrolyte.

XRD patterns of all typical electrolytes are characterized and displayed in Figure 1e. As the proportion of Si NPs increasing, the intensity of (111), (220), and (311) peaks increase significantly compared with the pure Si NPs (Figure S3, Supporting Information). In addition, the TGA curves and their corresponding detailly calculated results (Figure 1f and the interpolation table) show that the Si NPs proportion of 5%Si‐PEO_12_, 10%Si‐PEO_12_, and 15%Si‐PEO_12_ is relatively consistent with the actual design amount considering the oxidation of Si NPs. As displayed for the Si 2p spectrum in Figure 1g, the similar Si—Si bond in 99.0 eV^[^ ^30^ ^]^ and the Si—O bond in 103.1 eV^[^ ^31^ ^]^ were found on the 10%Si‐PEO_12_ and 15%Si‐PEO_12_. However, their strength is weaker than Si NPs. This result indicates the simple mixing did not change the surface oxidation of Si NPs, nor did generate new surface species.

Electrochemical stability against high voltage and Li anode is a crucial parameter to ASSLBs. To evaluate the electrochemical stability of SEs against high voltage, the linear sweep voltammetry (LSV) is adopted to investigate the electrochemical window at 60 °C and the results are shown in Figure 1h. In contrast to the PEO_12_ and 5%Si‐PEO_12_ electrolyte in which oxidation occurs at ≈3.75 V, the 10%Si‐PEO_12_ and 15%Si‐PEO_12_ electrolyte could maintain a high oxidative stability below 4.5 V. This indicates that the filling of Si NPs could stabilize the SE/Li interface and widen the electrochemical window, which is necessary for the working of high voltage batteries. Figure 1i shows the ionic conductivity and Arrhenius plots of all typical electrolytes. The detail EIS curves at 20–80 °C and the electrolyte thickness are shown in Figures S4 and S5, Supporting Information, respectively. A small quantity of Si NPs filling (5%Si‐PEO_12_) can improve the ionic conductivity at any temperatures, but filling with high content will decrease the ionic conductivity at high temperature due to the amorphous PEO ion conduction region occupied by inactive filler.^[^ ^11^, ^18^ ^]^ The ionic conductivity of 15%Si‐PEO_12_ at 60 °C is 3.92×10^−4^ S cm^−1^, which is slightly lower than other samples. The activation energy of 15%Si‐PEO_12_ is the lowest at 0.56 eV, which indicates that the addition of Si NPs is favorable for increasing the disorder degree of PEO, and thus improve the Li^+^ transfer behavior and reduced the activation energy.^[^ ^32^ ^]^

Li‐ion transfer number (*τ*_Li_^+^) is recognized as another important parameter for SEs, which is adopted to quantify the transport efficiency of Li^+^. Figure S6, Supporting Information, presents the polarization curves and AC impedance spectra of all typical electrolytes at 60 °C, and the results are summarized in Figure 1j. It can be seen that the introduction of a bit Si NPs can greatly increase *τ*_Li_^+^. However, 10%Si‐PEO_12_ and 15%Si‐PEO_12_ with higher content present a decreasing trend that may be related to the abundant hydroxyl group on the surface of Si NPs. The higher *τ*_Li_^+^ reflect there are more fleeter Li^+^ and immobilized anions.^[^ ^33^ ^]^ The hydroxyl group could adsorb Li^+^ and making more free TFSI^−^ ions, leading to decreased *τ*_Li_^+^. The higher ion conductivity and *τ*_Li_^+^ are conducive to alleviate the polarization effect so as to ensure long‐cycling stability for battery.^[^ ^33^, ^34^ ^]^ Meanwhile, all the stainless steel (SS)/all typical electrolytes/SS cells exhibit similar steady current density in a DC polarization of 100 mV (shown in Figure S7, Supporting Information), indicates that the addition of Si NPs does not significantly change the electronic conductivity of composite electrolyte.

**Figure 2:**
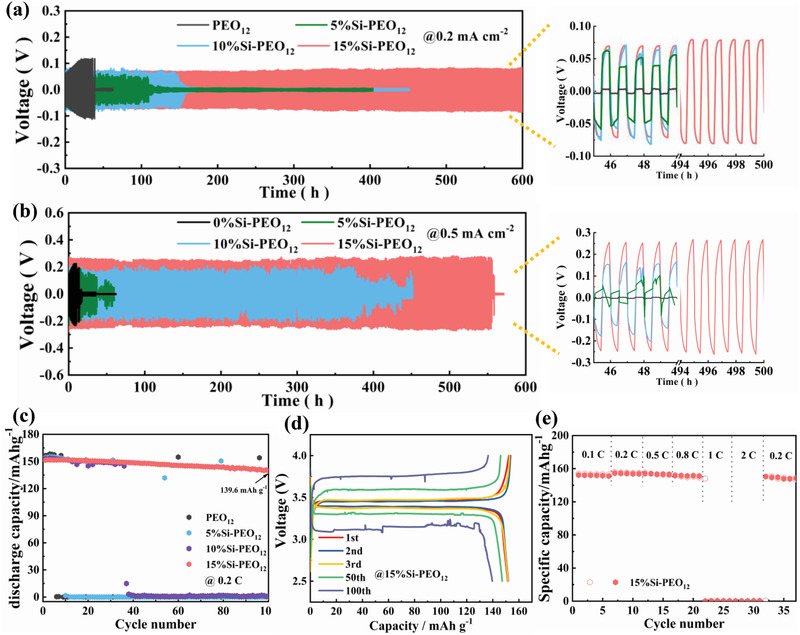
Cycling performance of Li/SE/Li batteries by using all typical electrolytes with current density of a) 0.2 mA cm^−2^/0.1 mAh cm^−2^ and b) 0.5 mA cm^−2^/0.25 mAh cm^−2^, the right images show the enlarged voltage profiles at typical periods. c) Cycling performance of LFP/SE/Li batteries by using all typical electrolytes at 0.2 °C. d) The charge–discharge curves and e) rate performance of LFP/15%Si‐PEO_12_/Li batteries. All the batteries were testing under a temperature of 60 °C.

To further test the stability of Si‐PEO based solid electrolyte against Li, the Li plating/stripping curves at 0.2 mA cm^−2^/0.1 mAh cm^−2^ and 0.5 mA cm^−2^/0.25 mAh cm^−2^ were examined by galvanostatic discharge/charge voltage profiles in symmetric Li/SE/Li cells, as presented in **Figures** **2**a and 2b, respectively. In contrast to other cells, the Li/15%Si‐PEO_12_/Li cell presents a stable Li deposition without short circuit over 600 h at 0.2 mA cm^−2^, and a low overpotential of just 83 mV. According to the decreased *τ*_Li_^+^ of high Si NP content in SE (Figure 1j), it is obviously difficult to explain such significant improvement on Li plating/stripping performance from view of ion transport ability. We speculate that this phenomenon may be associated with the lithiation of Si NPs. The solid electrolyte at the SE/Li interface is usually subjected to the non‐uniform stress and electric field because of the discontinuous and uneven morphology of Li deposition, which would also make partial Si NPs inside SEs directly contact with the deposited Li. Therewith, the lithiation of Si NPs happened, which may swallow the protruding Li and smooth the interface, and thus greatly delay the time of electrolyte penetration. The Li dendrites “swallowing” effect is furthered confirmed under the deposition at higher current density. At a current density of 0.5 mA cm^−2^, the cells of Li/15%Si‐PEO_12_/Li short‐circuit at ≈555 h, while Li/10%Si‐PEO_12_/Li persists more than 400 h unexpectedly in comparison to the just ≈150 h of 0.2 mA cm^−2^. The higher current density means more uneven Li deposition, so the lithiation of Si NPs could be easier to carry out than the lower current density, thus Li/10%Si‐PEO_12_/Li cells can persist much longer than 0.2 mA cm^−2^.^[^ ^35^ ^]^ This unexpected deposition phenomenon at high current density further confirms our hypothesis of the lithiation for Si NPs. The introducing of SiO_2_ or Si nanoparticles coating onto the polypropylene (PP) separator has been demonstrated an effective way to regulate the Li deposition by the lithiation reaction in liquid electrolytes, and called as “dendrite‐eating” strategy.^[^ ^36^, ^37^ ^]^ However, such strategy have never been successfully adopted in SEs to resolve the Li dendrite.

The ASSLBs with using LFP cathode are assembled to further demonstrate the potential of Si‐based solid electrolyte. As shown in Figure S8, Supporting Information, the appropriate Si NP additive can help to increase the initial Coulomb efficiency (ICE) slightly, which may be due to the reduction of interfacial reaction. As displayed in Figure 2c, the LFP/15%Si‐PEO_12_/Li can still maintain a high specific capacity of 139.6 mAh g^−1^ and a capacity retention ratio of 92.0% after 100 cycles at 0.2 C compared to 3rd cycle. However, LFP/5%Si‐PEO_12_/Li and LFP/PEO_12_/Li cells go straight to ≈0 mAh g^−1^ in less than 10 cycles, and even LFP/10%Si‐PEO_12_/Li just last less than 40 cycles. Figure S9, Supporting Information, shows the charge–discharge curves of all cells at selective cycles for further revealing the failure cause. LFP/15%Si‐PEO_12_/Li can run 100 cycles stably with much lower polarization comparing to other cells, which all present a significant polarization and abnormal charging profiles in the first few cycles. The contradiction between the accumulation of current and the barrier of ion transport could aggravate the interface deterioration and enhance polarization, which is the key reason for the interface failure of ASSLBs.^[^ ^38^, ^39^ ^]^ In the charge state, the neutralization of electrons and ions is prerequisite to maintain stable potential. The abnormal charge phenomenon for LFP/PEO_12_/Li, LFP/5%Si‐PEO_12_/Li and LFP/10%Si‐PEO_12_/Li cells indicates that the interface of SE/Li is deteriorated seriously and even have obvious dendritic puncture. The rate performance of LFP/15%Si‐PEO_12_/Li cells is tested and shown in Figure 2e. When running at lower than or equal to 0.8 C, the battery performance is fine, but it fails quickly at high values of 1 and 2 C. This may be due to the low activity of Si NPs for ionic conductivity and ion transfer number.

**Figure 3:**
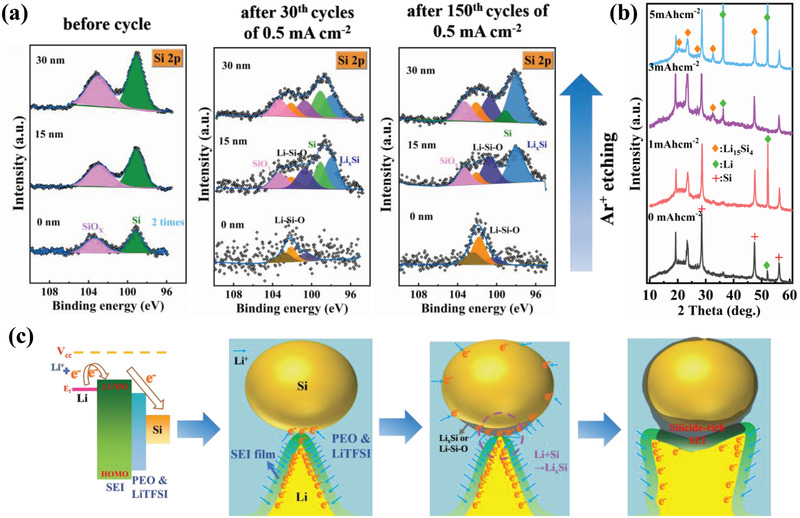
a) In‐depth XPS analysis of Si 2p spectra of 15%Si‐PEO_12_, and b) the XRD patterns with different depositing capacity. c) Schematic diagram of a multi‐interface space charge layer and their reactions.

The morphology analysis of the SE surface after Li plating/stripping cycle is carried out to further confirm the role of Si NPs in ion transport and blocking Li dendrites in SEs. Figures S10a and S11, Supporting Information, show the SEM images of 15%Si‐PEO_12_ and 5%Si‐PEO_12_ electrode after cycling. Some stacked Li dendrites are found on the surface of 15%Si‐PEO_12_ surface after 150 cycles, while 5%Si‐PEO_12_ electrode are covered by lots of spicules after 30 cycles. Some Si NPs still can be observed (Figure S10b, Supporting Information), where the particles in the damaged area are significantly larger than the particles in the non‐damaged area. Such larger particles should be assigned to Li‐Si compound. This means that the Si NPs can react with Li or some of the by‐products of Li, resulting to the growth of particles, and thus eliminating the original dendritic. To further investigate how the Si NPs change at the interface, XPS deep‐etching shown in **Figure** **3**a is adopted to detect changes in the Si composition. Compared with the uniform Si‐valence for the uncirculated 15%Si‐PEO_12_, Li—Si—O components (including Li_4_SiO_4_, Li_2_SiO_3_, and Li_2_Si_2_O_5_ species which divided into three peaks around at 100.7, 102.1, and 102.7 eV, respectively^[^ ^40^ ^]^) appear on the 15%Si‐PEO_12_ surface after 30 cycles, Li*_x_* Si species at 98.0 eV^[^ ^41^ ^]^ and Li—Si—O components are also found inside after further etching. Moreover, the co‐existence of Si and SiO_x_ indicate that the Si NPs inside the electrolyte are not fully lithiated, but partially lithiated. The result of deep etching to 30 nm also shows that the ratio of Li_x_Si and Li—Si—O is reduced, indicating that the lithiated degree is limited. With the increase of cycling (150 cycles), the depth of Si lithiated is further enhanced, which means that the Si NPs at the interface participate in the subsequent process. This is further confirmed by the XPS of the electrolyte after 300 cycles and the sample with initially depositing 8 mAh cm^2^ Li to one side (shown in Figure S12, Supporting Information). So we can determine that Si NPs can react with Li dendrite. In addition, the XRD patterns for 15%Si‐PEO_12_ initially depositing 0, 1, 3, 5 mAh cm^−2^ Li to one side were also tested to further confirm the composition of the product. As displayed in Figure 3b, the peak enhancement of Li_x_Si increased with the deposition amount indicates that the lithiation of Si NPs is indeed produced.^[^ ^42^, ^43^ ^]^

During Li deposition, the Li dendrites that infiltrate the electrolyte are filled with electrons. Large curvature means high current density, and the adsorption and diffusion of Li^+^ could be faster in these places.^[^ ^44^ ^]^ Theoretically, it is almost impossible for Si NPs to react spontaneously without directly contacting Li, because the Li dendrite surface is coated by an insulating but ionizing SEI film. However, as shown in Figure 3c, a multi‐interface space charge layer is formed between the tip of Li dendrites/SEI film/PEO&LiTFSI electrolyte/Si NPs. If Li^+^ is not enough to neutralize the electrons, the energy provided by the electrons is enough to make the electrons cross the energy barrier and reach the Si NP surface. Since the energy of Li^+^ reacting with Si after gaining electrons is less than that of metal Li deposition, so the Si NPs absorb the transferred Li^+^ preferentially, and generate Li—Si—O and Li*_x_* Si at the contact place.^[^ ^45^, ^46^ ^]^ The size of Si NPs increased after reactions, and then produced forces on the sharp Li dendrites, which causes the SEI film to be destroyed and direct contacts between Li and Si. Then, the spontaneous reaction occurred between Li and Si, intensifying the interface lithiation of Si and changing the original morphology of sharp dendrite. However, due to the reconstruction to silicide‐rich SEI film and the disappearance of the Li tip, a stable interface is formed between Li and Si NPs. In this way, Si NPs swallow and anchor dendrites, smoothing the Li tip, and thus remarkably alleviating the electrolyte puncturing by Li dendrite. In addition, we assemble all‐solid‐state batteries (Li/15%Si‐PEO_12_/Si anode) to examine the lithiation reaction of Si, as shown in Figure S13, Supporting Information, demonstrating the reversibility of Si anode in PEO‐based solid‐state batteries. Noteworthy, the de‐lithiated of Li*_x_* Si is harder than Li due to the higher de‐lithiated platform. That means that Li*_x_* Si is irreversible under the condition of excessive Li, but it could be reversible without enough Li. Besides, as displayed in Figure S14, Supporting Information, the results of in‐situ EIS analysis evaluate the role of the formation of conductive Li‐Si layer under the condition of high‐capacity Li metal deposition, and reveal the dependence relations between electronic conductivity, lithiation degree and battery short circuit.

**Figure 4:**
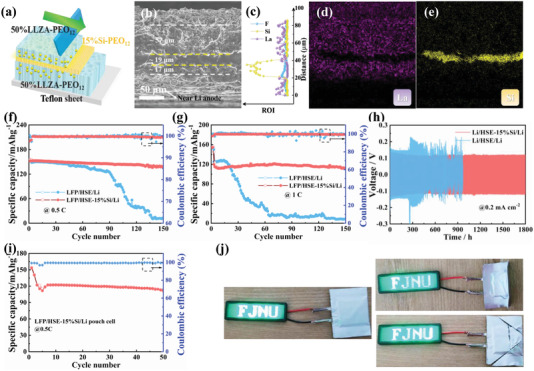
a) Schematic diagram of preparation process of sandwich solid electrolytes with HSE‐15%Si in the middle. b) Cross‐sectional SEM image of HSE‐15%Si. c) EDS line scanning image. EDS elemental mapping images of d) La and e) Si for HSE‐15%Si. Cycle performance of LFP/HSE/Li and LFP/HSE‐15%Si/Li batteries at f) 0.5 C and g) 1 C at 60 °C. h) Cycling performance of Li/HSE/Li and Li/HSE‐15%Si/Li batteries with current density of 0.2 mA cm^−2^ at 60 °C. i) Cycling performance of LFP/HSE‐15%Si/Li pouch cells at 0.5 C at 60 °C. j) The destructive condition test of LFP/HSE‐15%Si/Li pouch cells in folded and cut into pieces state.

The specialty of swallowing Li dendrite and possible reversibility let Si‐based solid electrolyte have great potential in practical application. However, the low Li^+^ diffusion and transfer number make it difficult to use directly, but it could be used as an additive interlayer in commercially available SEs. To demonstrate this application, 15%Si‐PEO_12_ as the interlayer to sandwich into the garnet‐type solid electrolyte, as depicted in **Figure** **4**a. The main body of 15%Si‐PEO_12_ is located in the middle but a few could be also found in the garnet‐type solid electrolyte due to the diffusion of slurry. Figure 4b shows the morphology of an asymmetrical sandwich structure, and the thickness of HSE‐15%Si interlayer is around 19 µm. EDS line scanning and mapping shown in Figure 4c–e reveals a clear separated layers. Then, it was assembled into all‐solid‐state LFP/HSE/Li cells with LFP cathode, and it should be mentioned that the 15%Si‐PEO_12_ interlayer was located near to Li anode side. The rate performance and the initial discharge–charge curves are shown in Figure S15, Supporting Information. The sandwich LFP/HSE‐15%Si/Li displays similar ICE and comparable capacity at low current density compared with LFP/HSE/Li, but the capacity at high current density (higher than 1 C) is a bit lower. This is mainly due to the low ionic conductivity and ion transport of Si‐based solid electrolyte.

**Figure 5:**
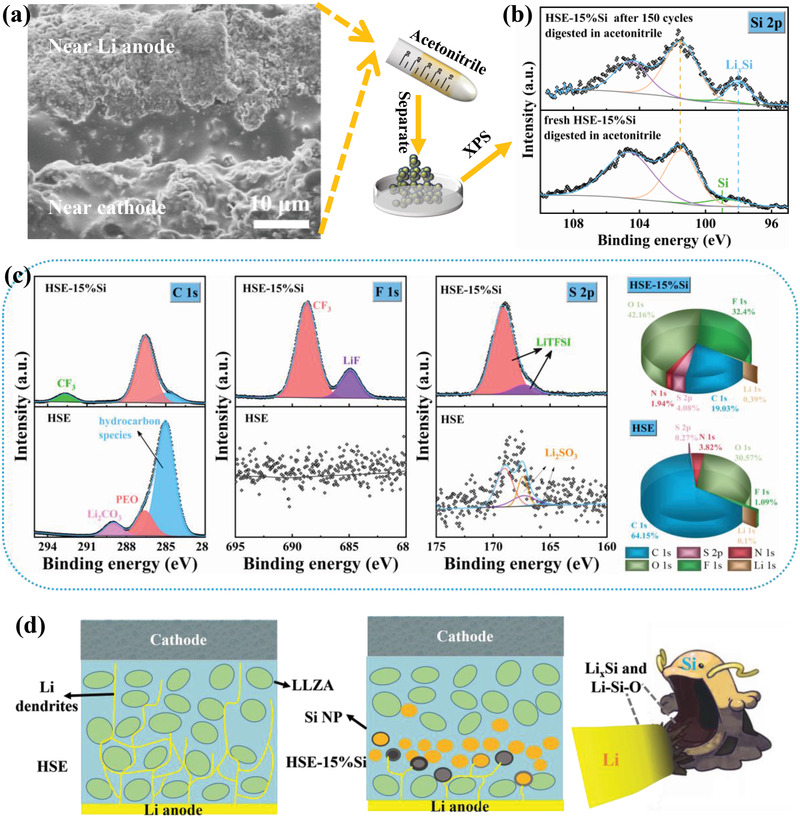
a) Cross‐sectional SEM image of HSE‐15%Si obtained from LFP/HSE‐15%Si/Li cells after 150 cycles of 0.5 C. b) The Si 2p spectrum of the separated powder for the HSE‐15%Si before and after 150 cycles obtained from dissolving in acetonitrile. c) XPS spectra for C 1s, F 1s and S 2p, and the total element ratio diagram for Li surface obtained from LFP/HSE/Li and LFP/HSE‐15%Si/Li cells after 150 cycles of 0.5 C. d) Schematic diagram of the mechanism for HSE and HSE‐15%Si.

Figures 4f and 4g display the cycling performance of HSE and HSE‐15%Si at different current density, respectively. Comparing to the LFP/HSE/Li cells where the capacity rapidly decays to 0 after 60 cycles, the LFP/HSE‐15%Si/Li cells still maintain a high specific capacity of 138.4 mAh g^−1^ with an average coulomb efficiency of 99.7% after 150 cycles, and a capacity retention of 90.8% compared to the 3rd cycle. At a high rate of 1 C, the capacity of LFP/HSE/Li decreases rapidly after less than 20 cycles, while the LFP/HSE‐15%Si/Li cells still maintain a stable specific capacity of 111.9 mAh g^−1^ after 150 cycles with a capacity retention of 93.3%. In addition, the EIS profiles after 150 cycles (Figure S16, Supporting Information, and the fitting results shown in Table S1, Supporting Information) reveal that the total length and depth of Li dendrites of HSE are much larger than that of HSE‐15%Si, which is deduced from the fact that the *R*_SEI_ of HSE (744.2 Ω) is much larger than that of HSE‐15%Si (321.7 Ω), and the *R_S_* (The bulk resistance of the solid electrolyte is related to thickness and properties) of 27.2 Ω is smaller than that of HSE‐15%Si (82.5 Ω). Furthermore, the ultrahigh *R*_ct_ of HSE also reflect structural damage of LFP cathode and electrolytes by the longitudinal growth of Li dendrites.^[^ ^46^ ^]^ The discharge–charge curves obtained from the different cycles (shown in Figure S17, Supporting Information) further prove these results. The results of Li–Li symmetric cells at 0.2 mA cm^−2^ are displayed in Figure 4h. In contrast to the failure after 400 h and high overpotential of HSE, HSE‐15% could maintain a stable electrochemical Li plating/stripping over 1800 h with a low overpotential of 115.0 mV. These results further reveal the role of the 15%Si‐PEO_12_ layer in regulating the stable Li deposition. To validate the practical application of HSE‐15%Si, the LFP/HSE‐15%Si/Li pouch cells (cell core: 4.0×3.0 cm) are configured and measured at 0.5 C at 60 °C. As shown in **Figure** **5**i, the pouch cell delivers a reversible discharge capacity of 111.1 mAh g^−1^ over 50 cycles at 0.5 C. Remarkably, the pouch cells can still light up a blue LED (FJNU patterns on it, shown in Figure 5j) with a strong glow even after folding and cutting into pieces, indicating the safety and reliability of such solid electrolytes for ASSLBs.

To further understand the effect of Si‐based solid electrolytes in anchoring and swallowing Li dendrite, the post‐cycle morphology and surface component analysis are necessary. Figure 5a shows the cross section morphology of HSE‐15%Si electrolyte after 150 cycles at 0.5 C. It can be clearly seen that the 15%Si‐PEO_12_ layer still exists. Notably, tiny cracks or voids by decomposition are found in the garnet electrolyte layer (near Li anode) above the 15%Si‐PEO_12_ layer, while the lower layer maintains a well gelatinous appearance similar to initial state. This suggests that the 15%Si‐PEO_12_ layer did indeed protect the solid electrolyte on the LFP side from electron tunneling induced by the decomposition of Li dendrite. In addition, the Si 2p spectra for the separated powder of HSE‐15%Si before and after 150 cycles are measured by dissolving in acetonitrile. As shown in Figure 5b, the extra pristine Li_x_Si peak compared to the pristine sample indicates that Si NPs did participate in the process of swallowing Li dendrites in the sandwich‐structure electrolyte.

The surface of Li anode and the SE near Li after cycling are tested and presented in Supporting Information. The HSE surface morphology shown in Figure S18a,b, Supporting Information, displays many large pores due to the decomposition of PEO caused by the large electric field on the Li dendrite. By the contrast, although the surface of HSE‐15%Si (Figure S18d,e, Supporting Information) is rough, it has relatively compact texture. In addition, as shown in Figure S18c,f, Supporting Information, the Li anode surface of HSE‐15%Si is relatively flat, compared with the lamellar and uneven surface topography of the Li anode obtained from HSE after 150 cycles, suggesting the remarkably improved homogeneous Li deposition between the HSE‐15%Si interface and Li anode. In order to further determine Li anode surface components after 100 cycles of 0.5 C, XPS measurement is adopted and the full spectrum results are shown in Figure S19, Supporting Information. As displayed in Figure 5c, five peaks around at 285.0, 286.6, 289.0, 292.7 eV are assigned to hydrocarbon species, PEO, Li_2_CO_3_, and —CF_3_ species, respectively.^[^ ^47^, ^48^, ^49^ ^]^ It can be seen that the Li surface of HSE has more Li_2_CO_3_, less CF_3_, and the composition of PEO is reduced, but the composition of hydrocarbon species is increased instead compared to HSE‐15%Si. This indicates that the SEI on the Li anode surface of HSE is thicker and the main component is the reaction product of residual H_2_O in SEs and Li, which also reflects the severious oxidation of HSE. That is also supported by the O 1s (Figure S20a, Supporting Information). Moreover, S 2p spectra display that the oxidation decomposition of HSE surface is very serious from the generation of Li_2_SO_3_. For the HSE‐15%Si, the CF_3_ and LiF together constitute the F 1s with a high proportion of 32.4% in all elements, indicating that the SEI film in HSE‐15%Si is thin and LiF occupies the majority. The surface composition of N 1s spectra also confirms this fact. It has been demonstrated that the SEI with abundant LiF is favorable for the stable Li deposition at the interface.^[^ ^50^ ^]^

The relative distance between the 15%Si‐PEO_12_ interlayer and Li anode has a great effect for the alleviating the Li dendrites. As shown in Figure S21, Supporting Information, the prepared LFP/Li cell using the symmetrical HSE‐15%Si with a sandwich structure of 31–19–29 µm just maintain 60 cycles at 1 C, which is much lower than the performance of the asymmetric HSE‐15%Si and comparable to the pristine HSE. This result suggests that the effect of eliminating Li dendrites needs the introduction of 15%Si‐PEO_12_ interlayer next to the Li anode, further conforming the Li dendrites inside the solid electrolytes are original from Li anode side and the dendrites swallowing mechanism by lithiation of Si. As shown in Figure 5d, in brief, LLZA particles can enhance the Li^+^ transfer ability and acts as a physical isolation to Li dendrite. While the Si‐based intermediate layer, like a phagocyte described in illustrations, can not only swallow the longitudinal Li dendrite, but also anchor Li dendrite until it changes from tip to plane. In the way, the main Li deposition process would be carried out on the Li anode surface, which avoids excessive electron–ion neutralization inside the electrolyte and ensures the smooth transfer channels of Li^+^. In addition, the introduction of Si‐based interlayer avoids the interface separation and the internal damage of SE caused by the lateral growth of Li dendrite also avoids the battery short‐circuit caused by the longitudinal growth and ensures the stability of the SE under high current density and long‐term cycle.

## 3 Conclusion

In summary, Si NPs filling into the PEO‐based solid electrolytes can effectively regulate the Li deposition at the interface and the growth inside the electrolytes through the lithiation effect. The irregular growth of Li dendrites could be controlled, and thus remarkably alleviating the by Li dendrites puncturing the solid electrolyte. This may be because the lithiated Si would swallow and anchor the Li dendrites in the electrolytes, leading to significantly improved cycle life of ASSLBs. Furthermore, such strategy was verified and adopted in the typical LLZA‐PEO solid electrolyte through constructing a sandwich structure by using Si‐PEO based electrolyte as a functional interlayer. Such sandwich solid electrolyte displays much improved cycling stability compared to common LLZA‐PEO electrolyte due to the stable interface structure by eliminating the irregular growth of Li dendrites, and thus reducing the damage of electrolyte film and short circuit. At last, the prepared pouch cell by the sandwich electrolyte exhibits comparable cyclic stability and is allowable for folding and even cutting into pieces. This novel and portable strategy is proven to be extremely effective and has high potential for practical ASSLBs.

## Conflict of Interest

The authors declare no conflict of interest.

## Supporting information

Supporting Information

## Data Availability

Research data are not shared.
