# Supplementary material for: Swallowing Lithium Dendrites in All‐Solid‐State Battery by Lithiation with Silicon Nanoparticles
Source: Adv Sci (Weinh). 2021 Nov 19;9(4):2103786. doi: 10.1002/advs.202103786 (PMC8811816; doi:10.1002/advs.202103786)
Supplement: Supplementary file 1 — Supporting Information [file ADVS-9-2103786-s001.pdf]

© 2021 Wiley-VCH GmbH

Supporting Information

for *Adv. Sci.*, DOI: 10.1002/advs.202103786

Swallowing Lithium Dendrites in All-solid-state Battery by Lithiation with Silicon Nanoparticles

Jianming Tao, Daoyi Wang, Yanmin Yang, Jiaxin Li, Zhigao Huang, Sanjay Mathur*, Zhensheng Hong*, Yingbin Lin*

Supporting Information

Swallowing Lithium Dendrites in All-solid-state Battery by Lithiation with Silicon Nanoparticles

Jianming Tao, Daoyi Wang, Yanmin Yang, Jiaxin Li, Zhigao Huang, Sanjay Mathur*, Zhensheng Hong*, Yingbin Lin*

1. College of Physics and Energy, Fujian Normal University, Fujian Provincial Solar Energy Conversion and Energy Storage Engineering Technology Research Center, Fuzhou,350117, China.
2. Institute of Inorganic Chemistry, University of Cologne, Greinstr.6, 50939 Cologne, Germany.
3. Fujian Provincial Key Laboratory of Quantum Manipulation and New Energy Materials, Fuzhou, 350117, China.
4. Fujian Provincial Collaborative Innovation Center for Advanced High-Field Superconducting Materials and Engineering, Fuzhou, 350117, China.

Experimental Section

Synthesis of $Li_{6.7}La_3Zr_2Al_{0.1}O_{12}$ particles: The garnet-typed LLZA micro-sized particles are prepared via a hydrogel method. Typically, 20 g of polyvinyl alcohol (PVA, M_w=47,000, Aladdin) is dissolved in 100 mL of deionized water by stirring at 80 ^oC for 4 h. After cooled down to room temperature, $LiNO_3$, $La(NO_3)_3\cdot6H_2O$ and $Al(NO_3)_3\cdot9H_2O$ with a stoichiometric ratio of 7.37(excess 10%): 3: 0.1 are dispersed into this solution by stirring 12 h. Subsequently, this solution is further cooled down to 3 ^oC, then 1.0 mL of glutaraldehyde (GA, 50% in H_2O, Aladdin) is added with stirring. After mixed for 15 min, the solution is casted onto an aluminum foil through a doctor blade of 2mm and dried at 80 °C for 3 h. Finally, the dried film was collected and subsequently calcinated at 800 ^oC for 5 h in air at a heating rate of 1 °C min^{-1} to gain $Li_{6.7}La_3Zr_2Al_{0.1}O_{12}$ (LLZA).

Synthesis of hybrid solid electrolyte film: The hybrid solid electrolyte based on Si nanoparticles is prepared by a solution casting method. Poly(ethylene oxide) (PEO, M_w= 400 000, Aladdin) and LiTFSI (Aladdin) are firstly dissolved in anhydrous acetonitrile with an $[EO]/Li^+$ ratio of 12, then the Si nanoparticles (~100 nm, Hefei Keller Nano Energy Technology Co., Ltd) with different weight ratios (a total weight of PEO, LiTFSI and Si

nanoparticles) are added to the above solution. After mechanically stir in dry air (humidity < 40%) for 12 h, the solution is degassed under 25 in Hg vacuum for 30 min, and then cast onto a teflon sheet and dry at 65 °C for 12 h under vacuum to obtain the Si-based solid electrolyte. In this paper, the hybrid solid electrolytes with 0 wt%, 5 wt%, 10 wt% and 15 wt% Si nanoparticles are marked PEO_{12}, 5%Si-PEO_{12}, 10%Si-PEO_{12} and 15%Si-PEO_{12}, respectively. The preparation process of hybrid solid electrolyte contained LLZA powder (named HSE) is similar to the Si-based electrolyte, except that the different amounts of Si nanoparticles are replaced with 50 wt% LLZA powder. The sandwich-structure (not symmetrical) solid electrolyte (named symmetrical HSE-15%Si) and sandwich-structure (symmetrical) solid electrolyte (named HSE-15%Si) is fabricated layer by layer using the same method. Specifically, a single layer of HSE slurry cast onto a teflon sheet, then 15wt% Si-PEO_{12} slurry after it dries, and then another layer of HSE slurry to obtain HSE-15%Si. Meanwhile, it ensures that the total thickness of HSE, HSE-15%Si, symmetrical HSE-15%Si is close to each other.

Materials characterization: The morphologies and microstructures of all the as-prepared samples are determined by field-emission scanning electron microscopy (FESEM; HITACHI, SU-8010) equipped with an energy-dispersive spectroscopy (EDS). X-ray Powder Diffractometer (XRD) patterns of all the as-prepared samples are obtained by Rigaku MiniFlex II using Cu K_α radiation (λ=1.5405 Å) in an angular range of 10°-60°. X-ray Photoelectron Spectroscopy (XPS) spectra is acquired with ESCALAB 250Xi (Thermo Scientific) with monochromatic Al Kα 1486.6 eV radiation, and the etched depth with an etching speed of ~3.2 nm min^{-1} to Si (~0.07 nm s^{-1} to the standard Ta_2O_5 in the instrument) under Ar^+-ion bombardment at 500 eV. Thermogravimetric (TG) analyses are conducted on a Netzsch STA449F3 analyzer from 25 ^oC to 800 ^oC with a temperature ramp of 5 ^oC min^{-1} under an air flow.

Electrochemical measurements: The ionic conductivity is measured by sandwiching the composite solid electrolyte between two stainless steel electrodes (SS|SE|SS, the area of SS is ~2 cm^2). The ionic conductivity plots are obtained by electrochemical impedance spectroscopy (EIS) with frequency range from 4 MHz to 1 Hz at the temperature from 20 to 80 °C to Zahner Zennium IM6 electrochemical workstation with an AC amplitude of 5 mV.

$$\sigma = \frac{L}{R_b\, S} \#\mathrm{S1}$$

where R_b is the bulk resistance obtained by the EIS, and L and S are the thickness and effective area of electrolytes respectively. The activation energy (E_a) of composite solid electrolyte is calculated by Arrhenius formula. Li|SE|SS cells are applied to test the electrochemical stability window through linear sweep voltammetry (LSV) between 2.5 V and 5.3 V (vs. Li^+/Li) at a scanning rate of 1 mV s^{-1} at 60 °C on CHI 1000C electrochemical workstation. Li|SE|Li cells are assembled for test the interfacial resistance and polarization under elevated current density. The lithium-ion transference number ($\tau_{Li}{}^+$) of the composite solid electrolytes is measured at 60 °C in a symmetric Li|SE|Li cells with a DC polarization voltage of 20 mV associated with the AC impedance measurement, and calculated following Eq. (2). The initial (I_0) and steady (I_{ss}) currents are obtained from a DC polarization test. R_0 and R_{ss} are obtained from the AC impedance measurement with the frequency between 4 MHz and 1 Hz representing the interface impedance before and after the test, respectively.

$$\tau_{Li^+} = \frac{I_{ss}(\Delta V - I_0 R_0)}{I_0(\Delta V - I_{ss} R_{ss})} \#S2$$

The polarization curves of Li|SE|Li cells are generated through galvanostatic charge-discharge at 0.2mA cm^{-2}/0.1mAh cm^{-2} or 0.5mA cm^{-2}/0.25mAh cm^{-2}on LAND 2001A battery testing system. The integrated all-solid-state Li battery is prepared using solid electrolyte, $LiFePO_4$ as cathode material, and Li metal as anode and sealed in 2032 coin cells. The $LiFePO_4$ cathode consists of 70 wt% LFP, 10 wt% PEO, 10 wt% LiTFSI, and 10 wt% carbon

blacks. The mass loading of $LiFePO_4$ in batteries is above 2.0-2.5 mg cm^{-2}. The batteries are assembled in glove box in Ar atmosphere without using separator or any liquid electrolyte. The cycling performance of all-solid-state lithium battery at 60 °C was acquired pre-cycle 2 times at 0.1 C and then cycle at other rate with voltage range from 2.5 to 4.0 V. The rate performances are obtained from 0.1 C to 2 C at 60 °C. To assess the electrochemical performances of HSE-15%Si in the practical cell, we fabricate simple pouch cells using $LiFePO_4$ as cathode and the Li foil with a 100 μm thickness as anodes. The weight ratio of $LiFePO_4$: carbon blacks : PVDF : HSE slurry is 85:5:5:5 in the cathode, and the mass loading is ~3 mg▪cm^{-2}. The dimension of the cathode in pouch cells is 3 cm in length and 4 cm in width, the anode dimension is 3.2 cm in length and 4.2 cm in width.

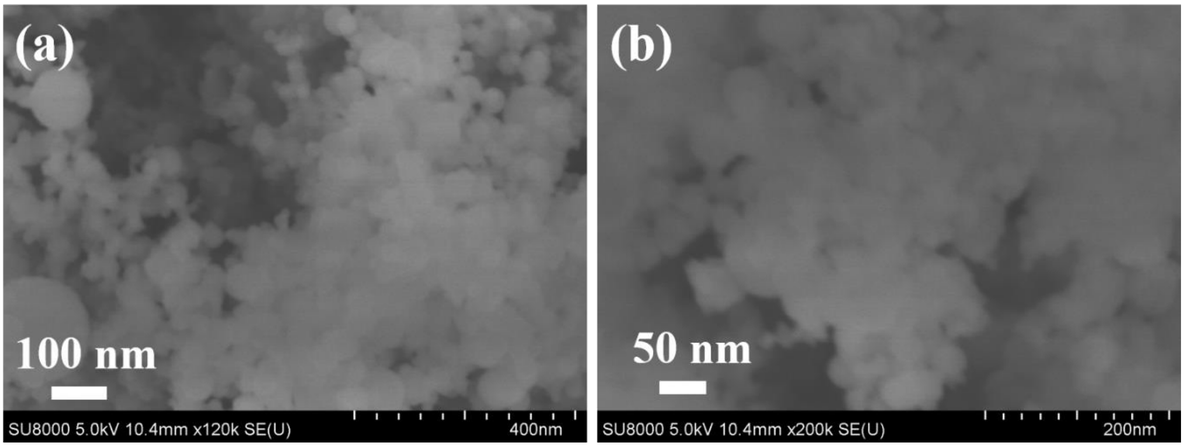

Figure S1 (a, b) SEM image of silicon nanoparticles.

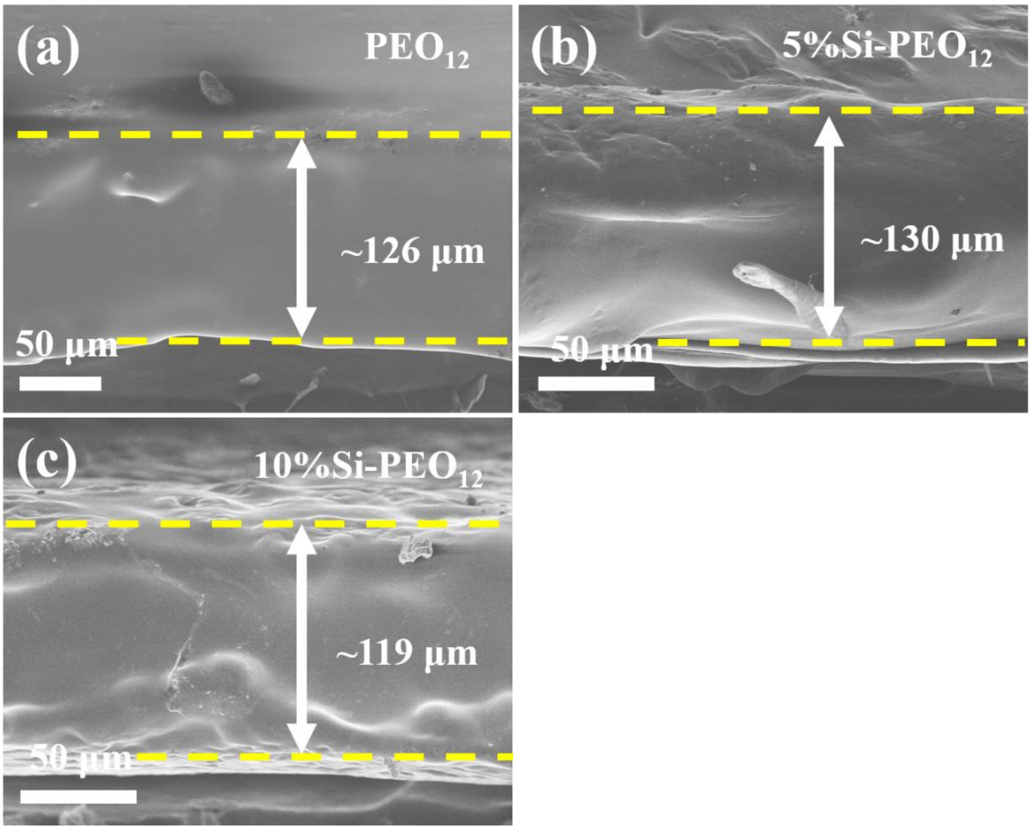

Figure S2 Cross-sectional SEM image of (a) PEO_{12}, (b) 5%Si-PEO_{12}, (c) 10%Si-PEO_{12}

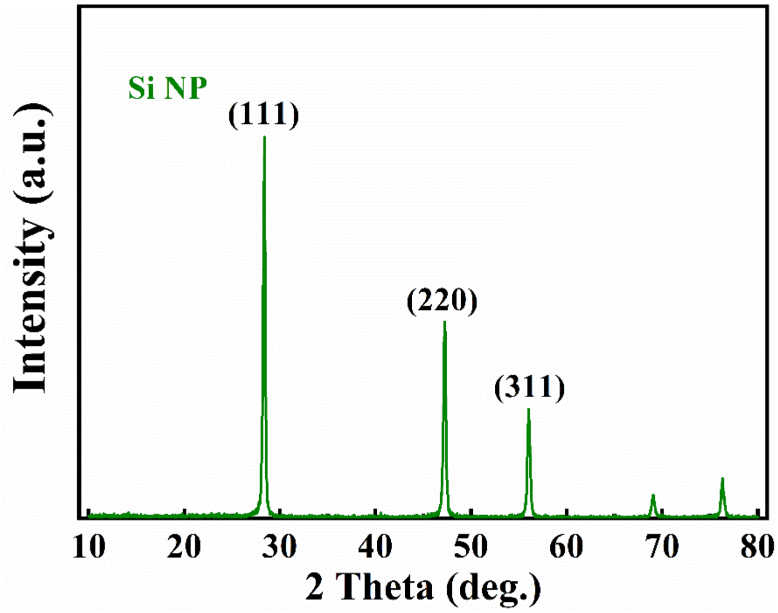

Figure S3 XRD pattern of silicon nanoparticles

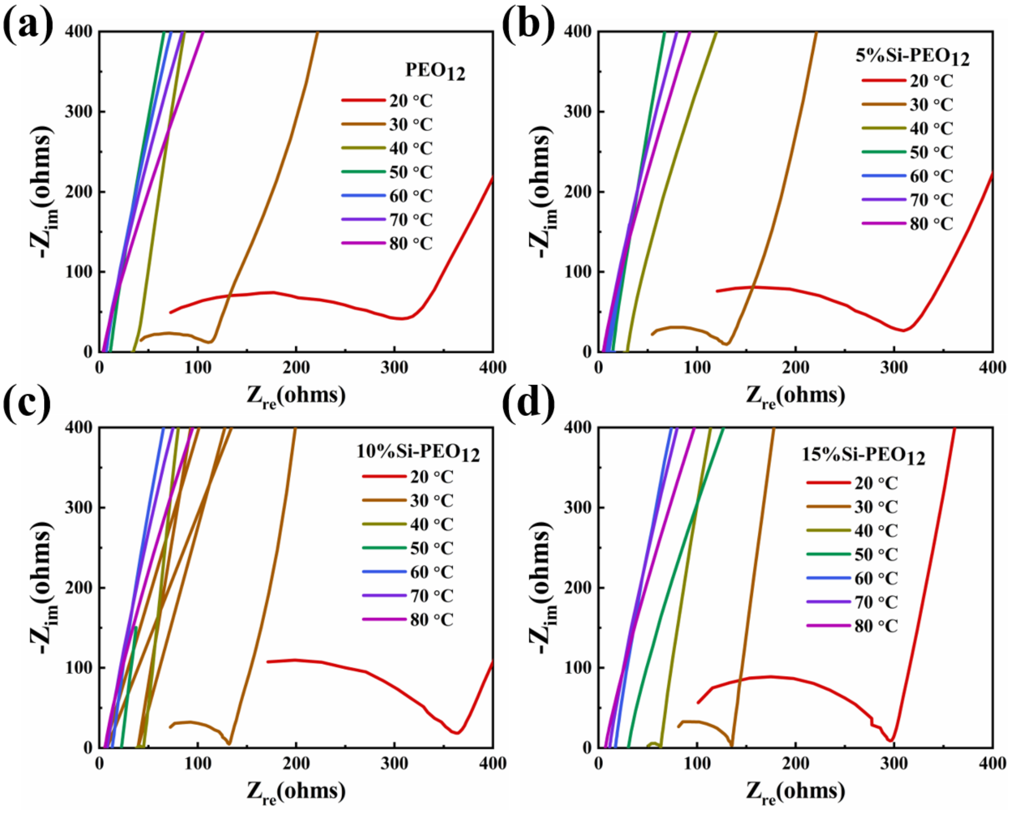

Figure S4 The impedance spectra of (a) PEO_{12}, (b)5%Si-PEO_{12}, (c) 10%Si-PEO_{12} and (d) 15%Si-PEO_{12} solid electrolytes sandwiched in two stainless steels form 20 °C to 80 °C.

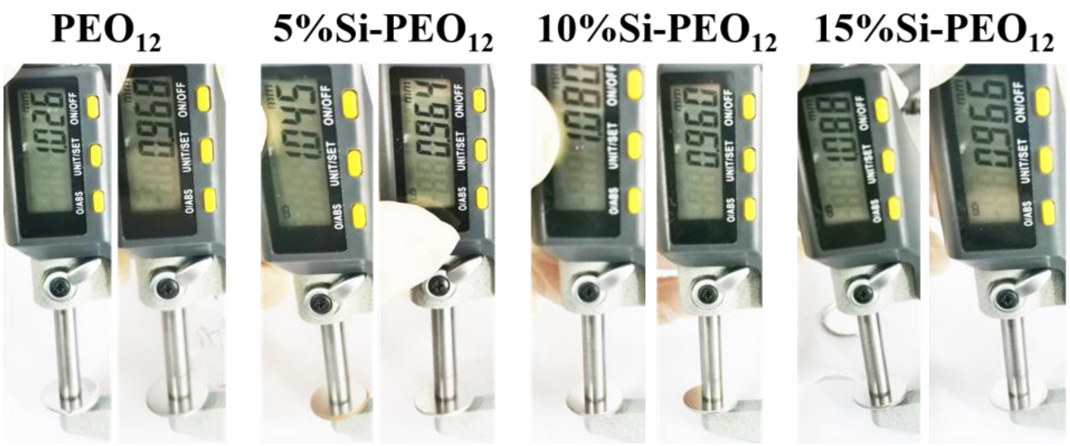

Figure S5 The thickness of SS/SE/SS and SS/SS without SE for PEO_{12}, 5%Si-PEO_{12}, 10%Si-PEO_{12} and 15%Si-PEO_{12}.

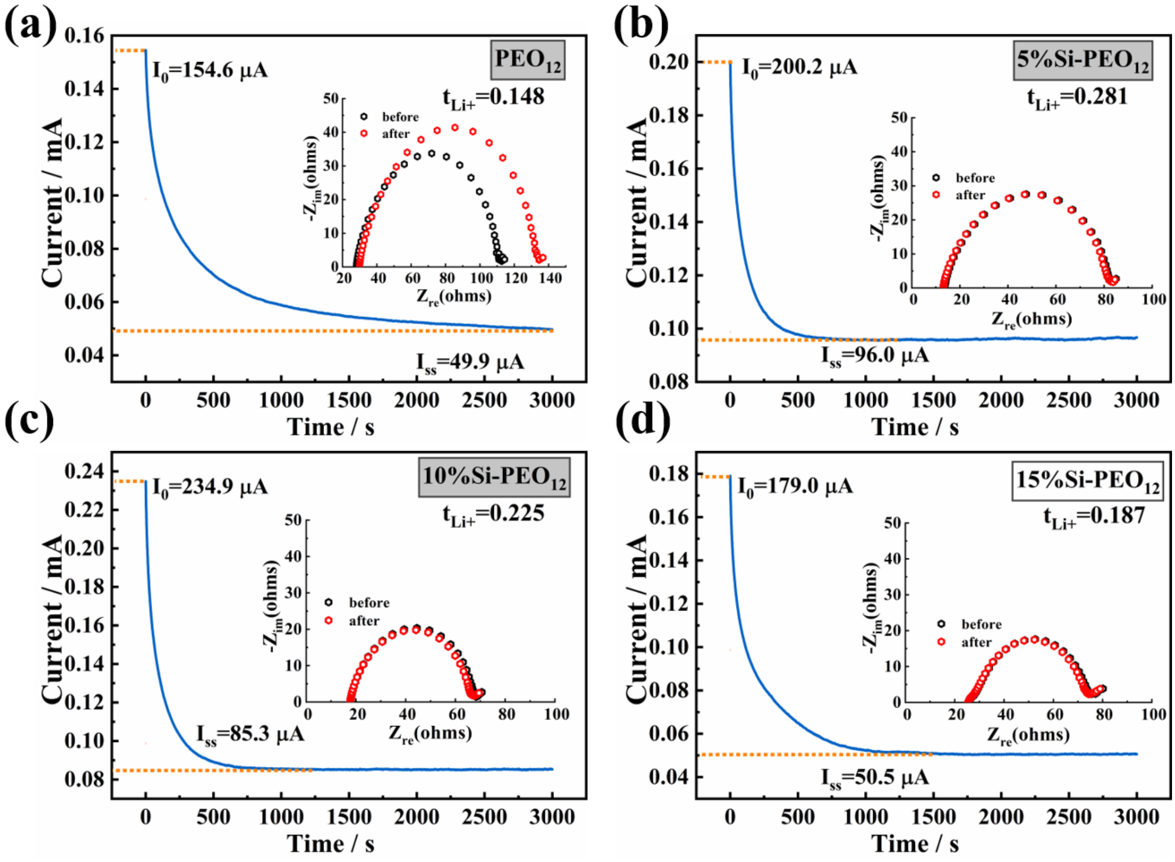

Figure S6 The polarization curves and Nyquist impedance spectra before and after polarization of (a) Li/PEO_{12}/Li, (b)Li/5%Si-PEO_{12}/Li, (c) Li/10%Si-PEO_{12}/Li and (d) Li/15%Si-PEO_{12}/Li cells.

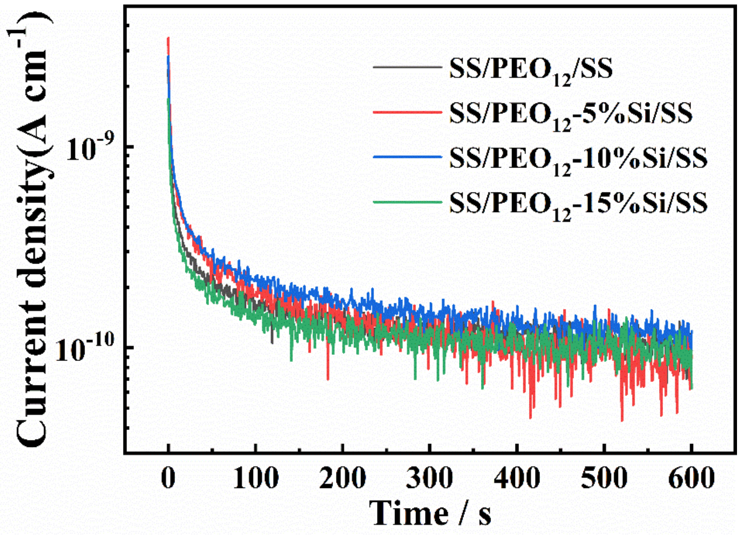

Figure S7 The current density-time curves of all the SS/Si-based electrolytes/SS cell at 60 °C. The current density is calculated using σU=U/ρ=I L/S, where ρ is resistivity, U is the test polarization voltage (100 mV), L is the thickness of solid electrolyte, the surface area of cell is S =2 cm^{-2}, the steady current is I obtained from the time-current curves.

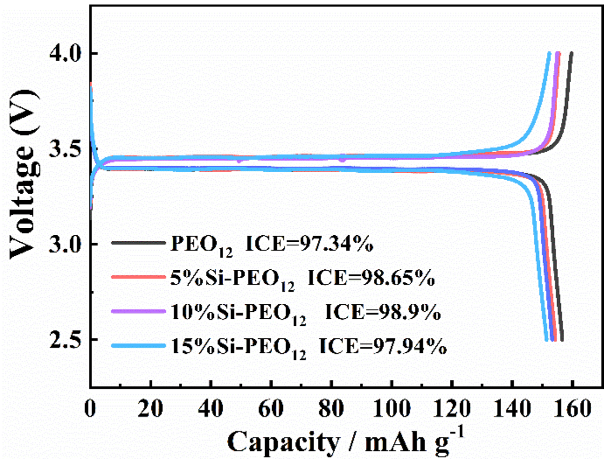

Figure S8 The initial discharge/charge curves for LFP/PEO_{12}/Li, LFP/5%Si-PEO_{12}/Li, LFP/10%Si-PEO_{12}/Li and LFP/15%Si-PEO_{12}/Li cells at 0.1 C.

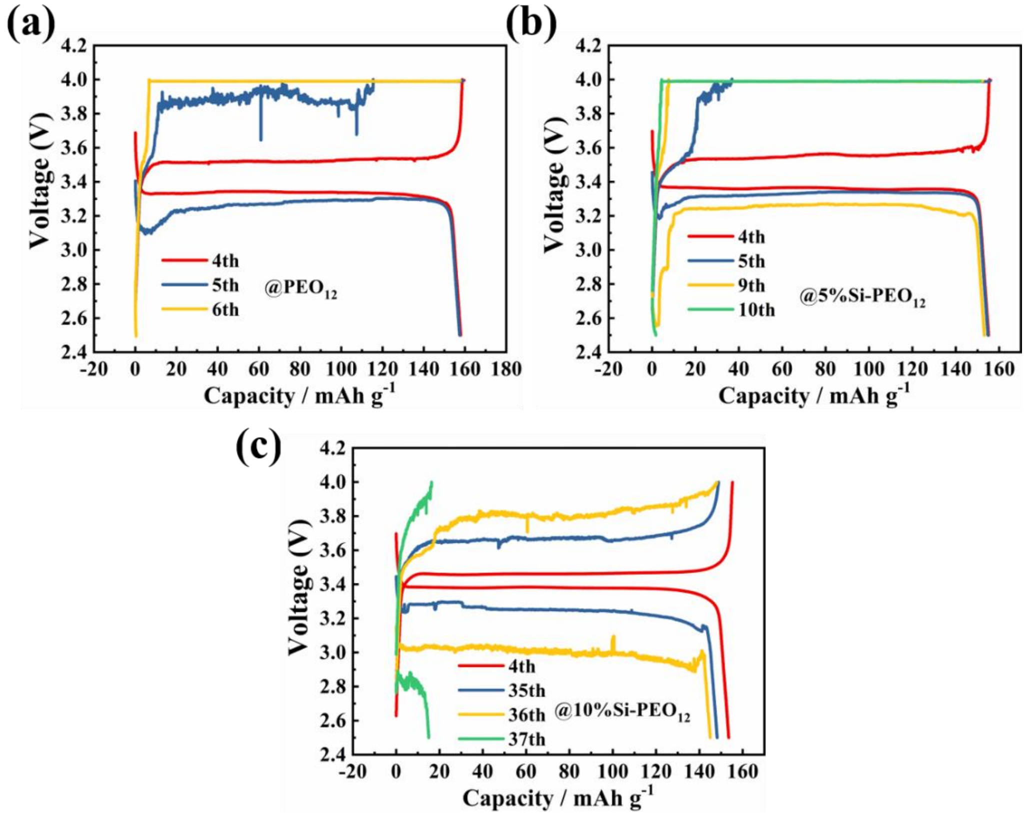

Figure S9 The discharge/charge curves after different cycles for LFP/PEO_{12}/Li, LFP/5%Si-PEO_{12}/Li, LFP/10%Si-PEO_{12}/Li and LFP/15%Si-PEO_{12}/Li cells at 0.2 C.

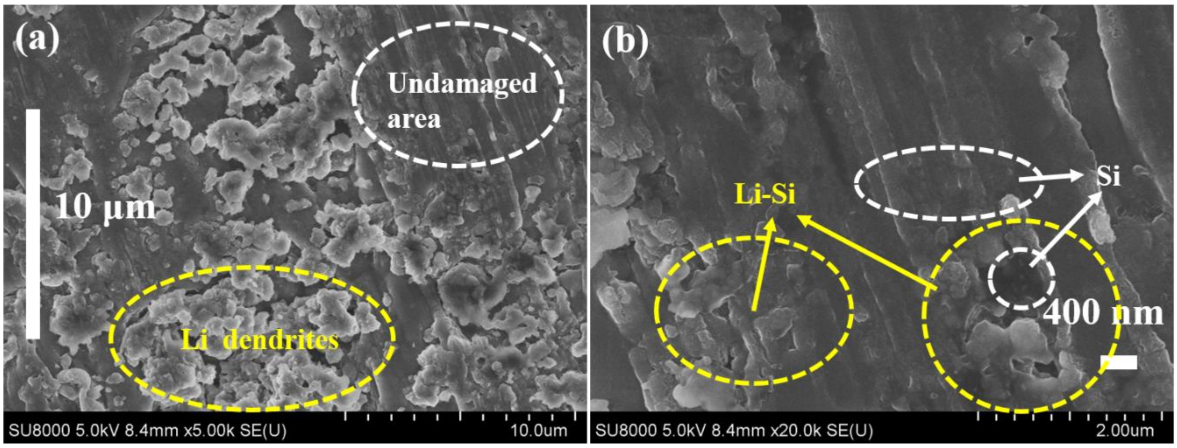

Figure S10 The surface morphologies of 15%Si-PEO_{12} electrode obtained from Li/15%-PEO_{12}/Li cells after 150 cycles of 0.5 mA cm^{-2}.

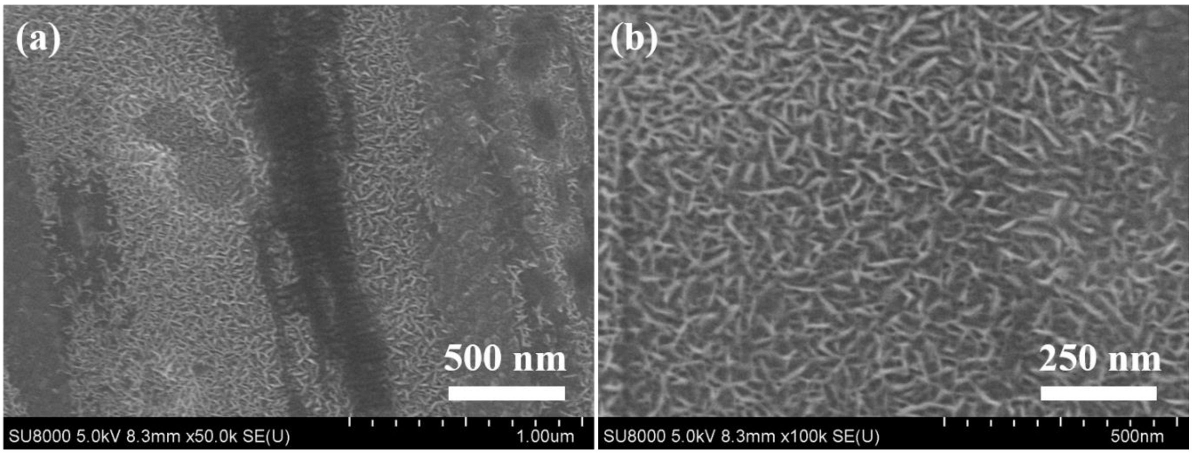

Figure S11 The surface morphologies of 5%Si-PEO_{12} electrode obtained from Li/5%-PEO_{12}/Li cells after 30 cycles of 0.5 mA cm^{-2}.

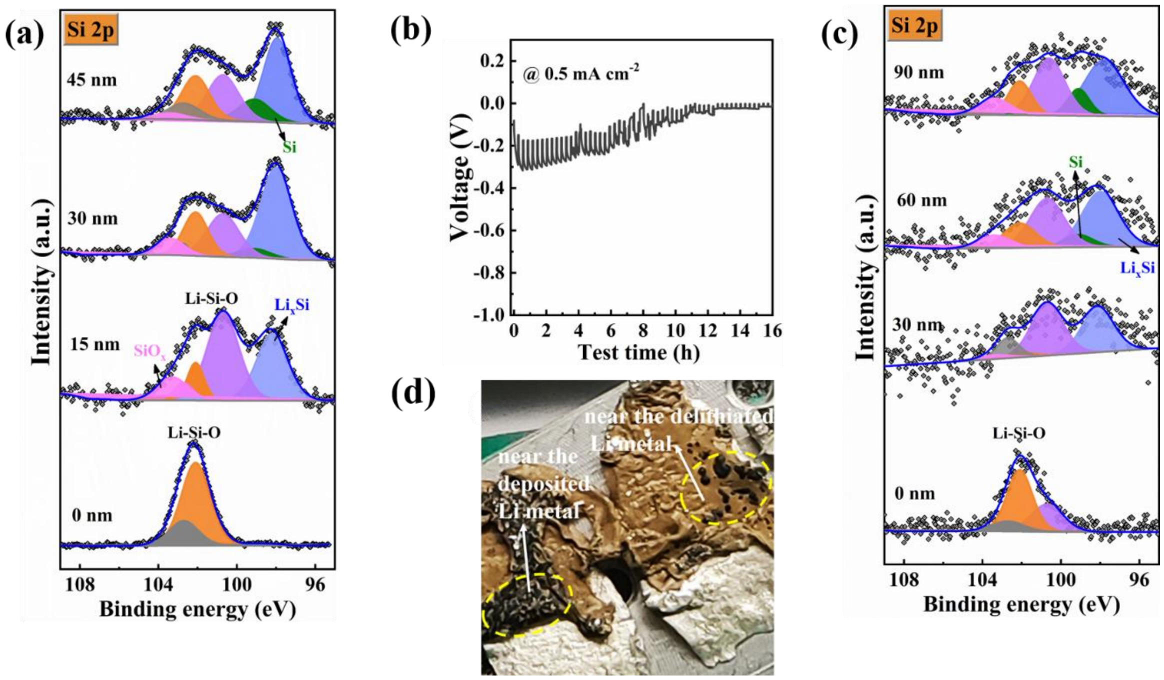

Figure S12 (a) In-depth XPS analysis of Si 2*p* spectra of 15%Si-PEO_{12} obtained from Li/PEO_{12}-15%Si/Li cells after 300 cycles of 0.5 mA cm^{-2}. (b) The voltage-time curves at 0.5 mA cm^{-2} of the Li/PEO_{12}-15%Si/Li cell. (c) In-depth XPS analysis of Si 2*p* spectra of 15%Si-PEO_{12} obtained from Li/ PEO_{12}-15%Si /Li cells after 16 h deposition at 0.5 mA cm^{-2}. (d) The photo-image of the electrolyte surface (One is near the deposited Li, the other one is near the delithiated Li) obtained from Li/PEO_{12}-15%Si/Li cell after 16h of 0.5 mA cm^{-2}.

To verify the existence of Li dendrites in long-term circulation and further prove the lithiation of Si NP, the Li/PEO_{12}-15%Si/Li cells were assembled and one-sided Li deposition was extremely performed at a current density of 0.5 mA cm^{-2} for 16 h. As shown in Figure S12b, the battery had completely short-circuited and the voltage was close to 0 V within 12 h. Meanwhile, the in-situ EIS tests performed every 15 min also revealed the fact of battery short- circuit. The XPS results (shown in Figure S12c, Si alloy peak can be found just a little at 60 nm after etching) show that the almost all the Si particles at the interface and near the interface are lithiated. Combining with the blackening (black Li-Si products by the lithiation with Si NP) on both sides, as shown in Figure S12d, it can be inferred that Li does grow into the electrolyte and eventually reaches the Li metal that is delithiated on the other side.

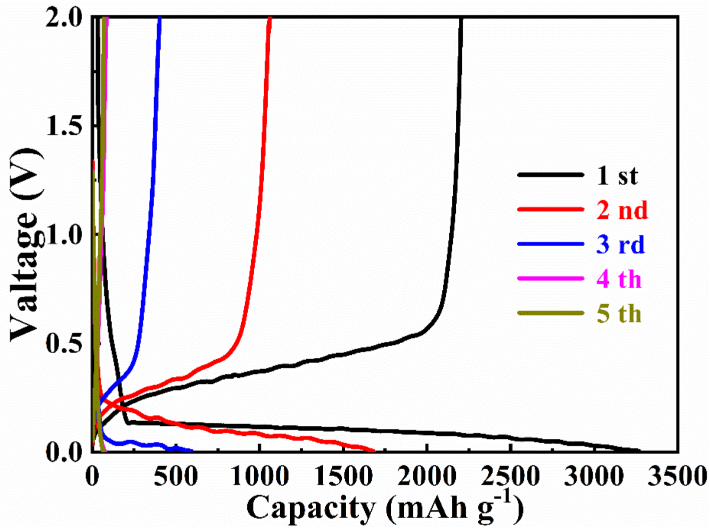

Figure S13 The discharge/charge curves after different cycles for Li/15%Si-PEO_{12}/Si cells at 100 mA g^{-1}.

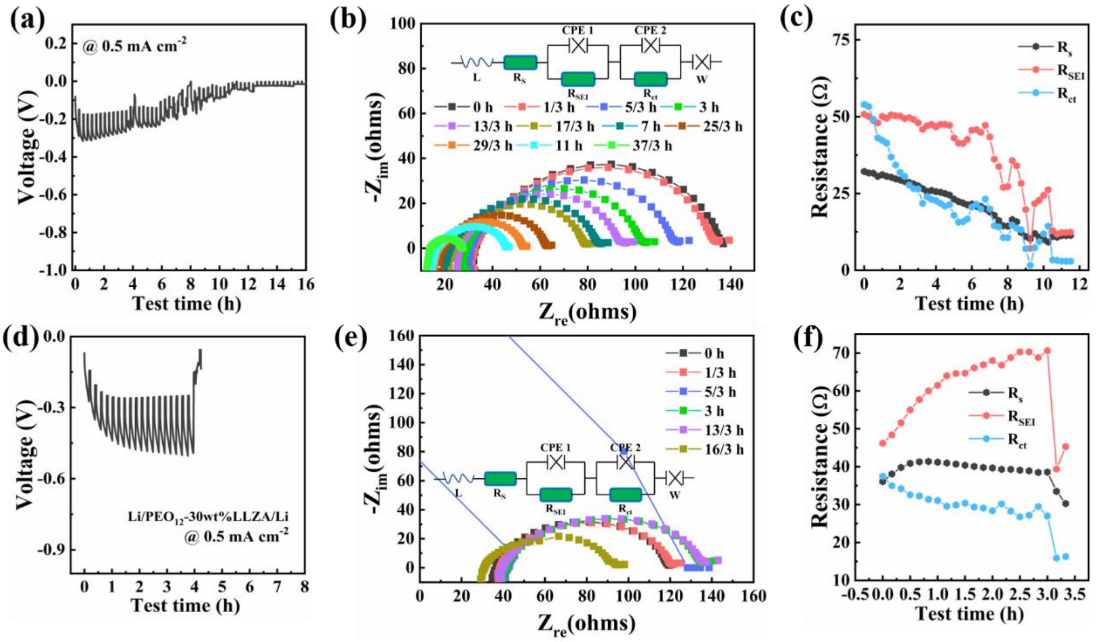

Figure S14 The voltage-time curves at 0.5 mA cm^{-2} for (a) the Li/PEO_{12}-15%Si/Li cell and (d) the Li/ PEO_{12}-30wt%LLZAO/Li cell. Nyquist plots after different deposition time at 0.5 mA cm^{-2} for (b) the Li/PEO_{12}-15%Si/Li cell and (e) the Li/PEO_{12}-15%Si/Li cell. The fitted results including R_s (bulk electrolyte resistance), R_{SEI} (the resistance of SEI film) and R_{ct} (the charge transfer resistance) for (c) the Li/PEO_{12}-15%Si/Li cell and (f) the Li/ PEO_{12}-30wt%LLZAO/Li cell. The preparation process of PEO_{12}-30wt%LLZAO electrolyte is similar to the Si-based electrolyte, except that the different amounts of Si nanoparticles are replaced with 30 wt% LLZA powder.

As shown in Figure S14a and d, compared with the batteries assembled by conventional solid electrolytes, Li/PEO_{12}-15%Si/Li batteries exhibit a higher upper limit of depositable capacity. The in-situ EIS analysis were tested in every 15 min in this process, corresponding Nyquist plots and fitting results were all shown in Figure S14. As displayed in Figure S14c, the bulk resistance of electrolyte (R_s) decreased slowly with deposition time, indicates that Li-Si layer formed does gradually improves the bulk conductivity. However, the electrons on Li-Si layer cannot be transferred from one silicon particle to another disconnected particle because the Si NP is dispersed in a ‘sea’ of polymers and does not form a connected network. This can also be verified by the SEI film resistance (R_{SEI}) remaining stable for nearly 7 h.

Because if electrons can jump from Li-Si layer to another Si NP, the growth of Li dendrite will be more rapid and the SEI film on its surface also be more and more, thus the R_{SEI} will rapidly become larger.

On this premise of low overall conductivity of electrolyte, the conductive Li-Si layer can better speed up the process of swallowing and convert Li dendrites and convert Li dendrites. Besides, the process is limited because the insulate SEI film formed at the contact space that "can cut" off electron transfer between the smoothed Li and Si NP, and thus reducing unnecessary Li loss. Of course, if large amounts of silicon have been converted into Li-Si layers (the battery is near short-circuited, that like the battery state after 7 hours deposition show in Figure S14a), the Li dendrites will grow more easily than before because of the decreasing electrical resistance as the lithiation of silicon in electrolyte. However, as we've shown, the above situation only happens in high-capacity lithiation/delithiation, and it's hard to do in low-capacity lithiation/delithiation. Because of this, the Li/15%Si-PEO_{12}/Li cell presents a stable Li deposition without short circuit over 600 h at 0.2 mA cm^{-2}, and the sandwich-structure solid electrolyte can demonstrate stable Li plating/stripping over 1800 h.

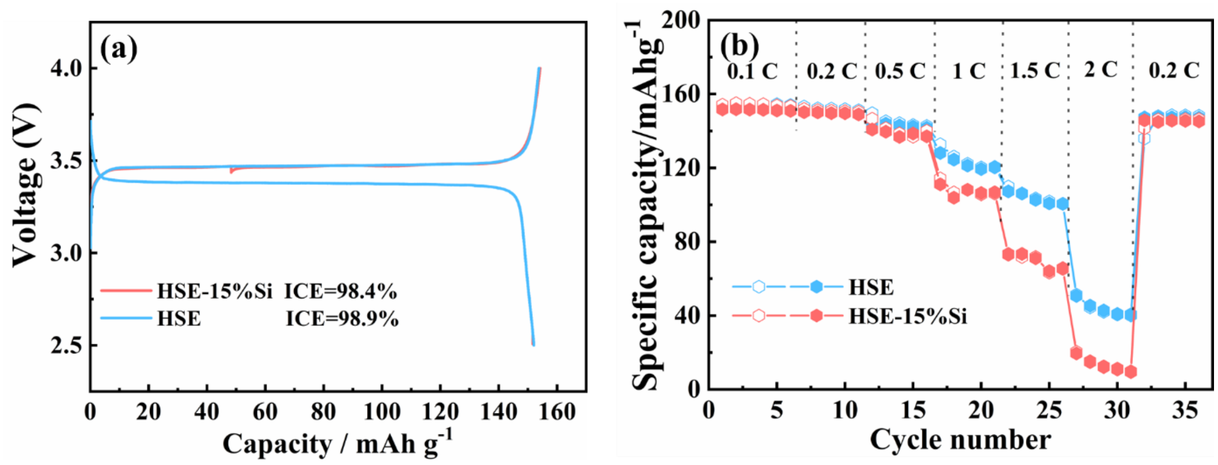

Figure S15 (a) The initial discharge/charge curves for LFP/HSE/Li, LFP/HSE-15%Si/Li cells at 0.1 C. (b) Rate capability of LFP/HSE/Li and LFP/HSE-15%Si/Li cells at 60 °C.

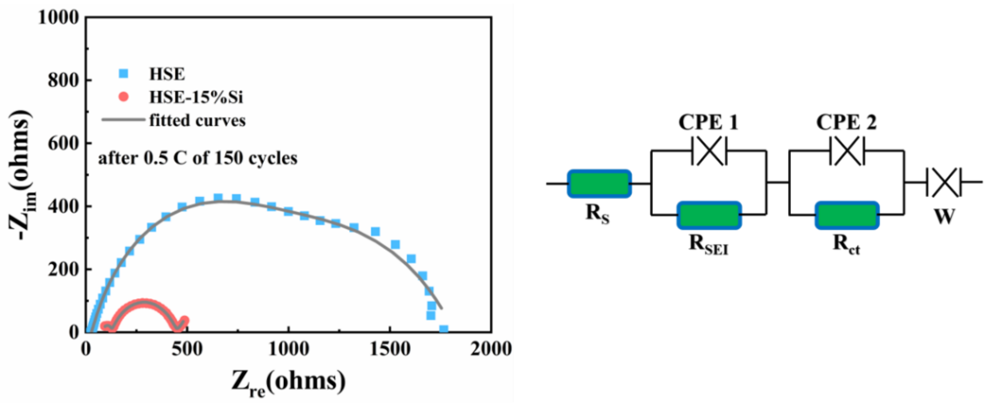

Figure S16 Nyquist plots after 150 cycles at 0.5 C for LFP/HSE/Li and LFP/HSE-15%Si/Li batteries.

Table S1

Summarized comparison of the electrolyte bulk resistance, the resistance of the SEI film, the charge transfer resistance by equivalent circuits.

Cells	R_s(Ω)	R_{ct}(Ω)	R_{SEI}(Ω)
**HSE after 150 cycles of 0.5 C**	27.2	1044.0	744.2
**HSE-15%Si after 150 cycles of 0.5 C**	82.5	43.1	321.7

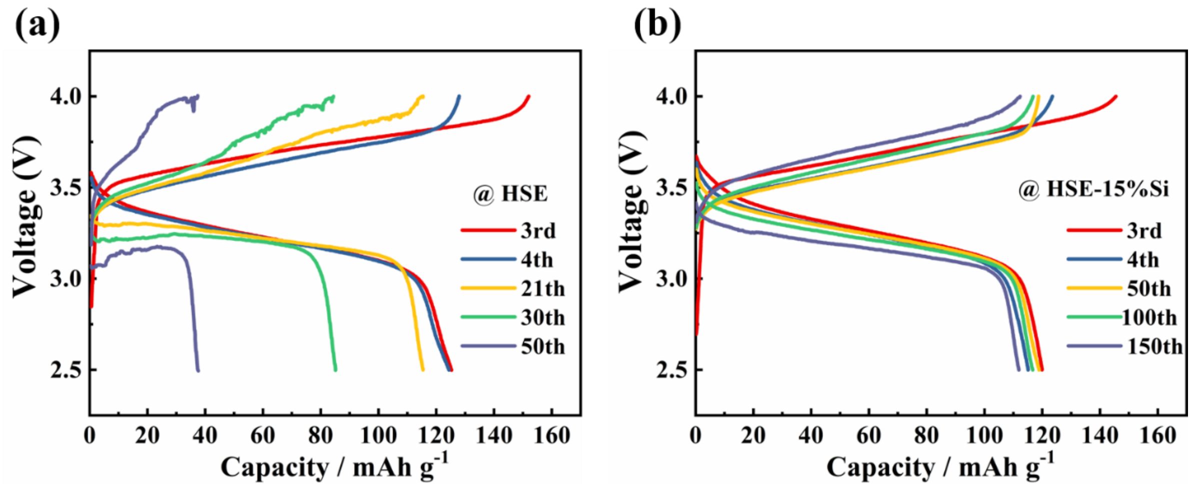

Figure S17 The discharge/charge curves after different cycles for LFP/HSE/Li, LFP/HSE-15%Si/Li cells at 1 C.

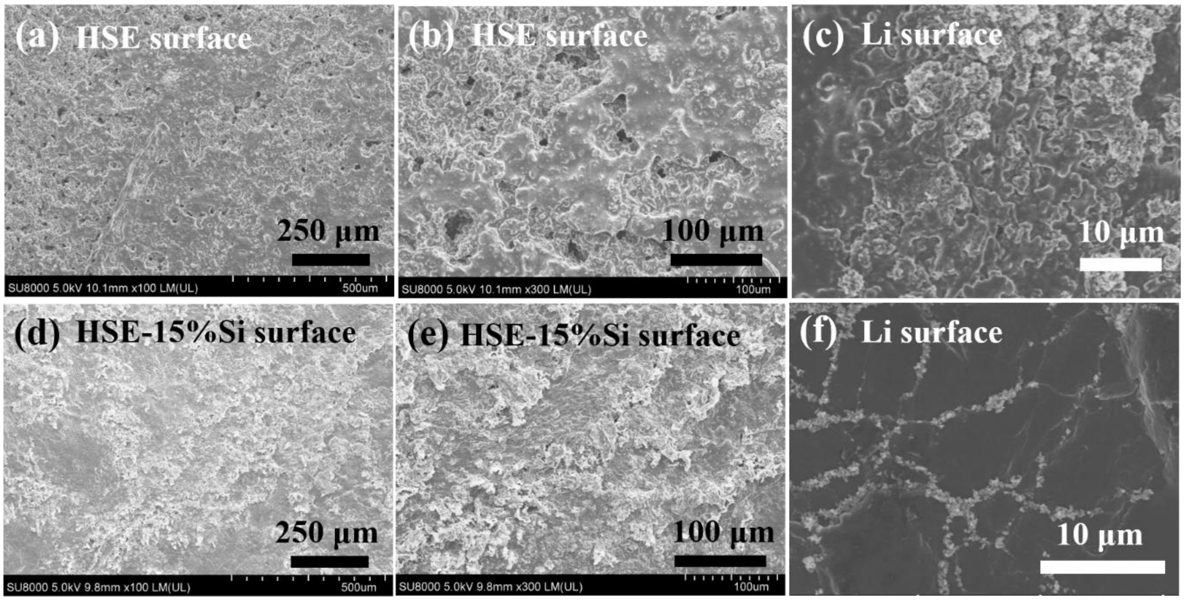

Figure S18 The surface morphologies of (a, b) HSE surface and (c) Li surface obtained from LFP/HSE/Li cells after 150 cycles of 0.5 C. The surface morphologies of (d, e) HSE-15%Si surface and (f) Li surface obtained from LFP/HSE-15%Si/Li cells after 150 cycles of 0.5 C.

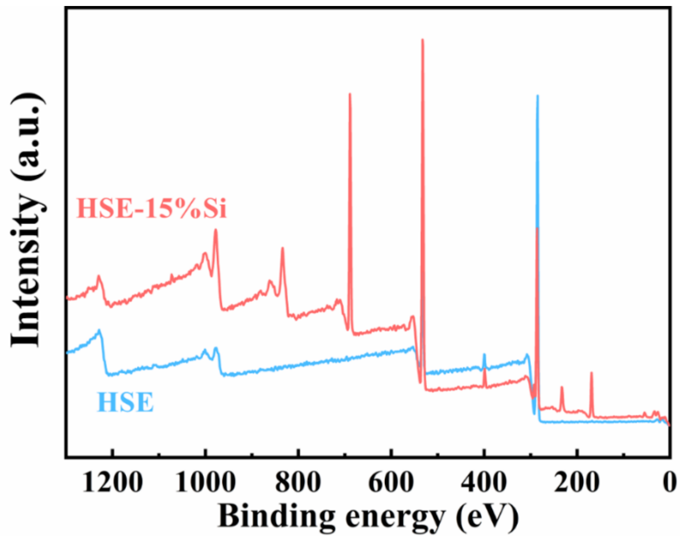

Figure S19 XPS survey spectrum for Li surface obtained from LFP/HSE/Li and LFP/HSE-15%Si/Li cells after 150 cycles of 0.5 C.

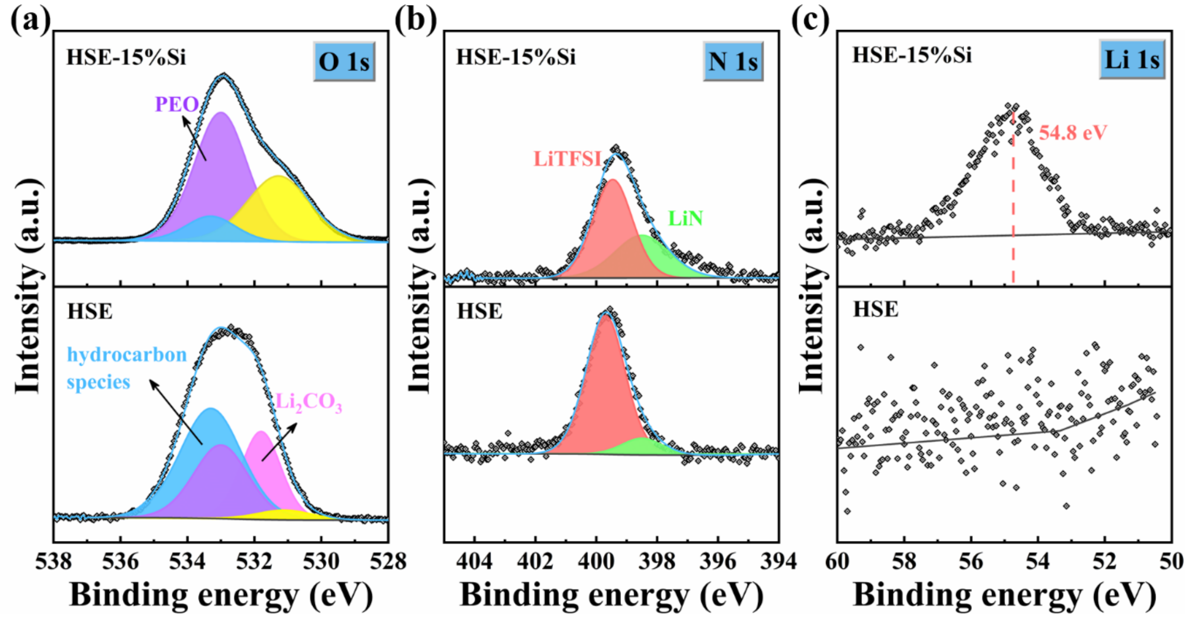

Figure S20 XPS spectra for (a) O 1*s*, (b) N 1*s*, (c) Li 1*s* for Li surface obtained from LFP/HSE/Li and LFP/HSE-15%Si/Li cells after 150 cycles of 0.5 C.

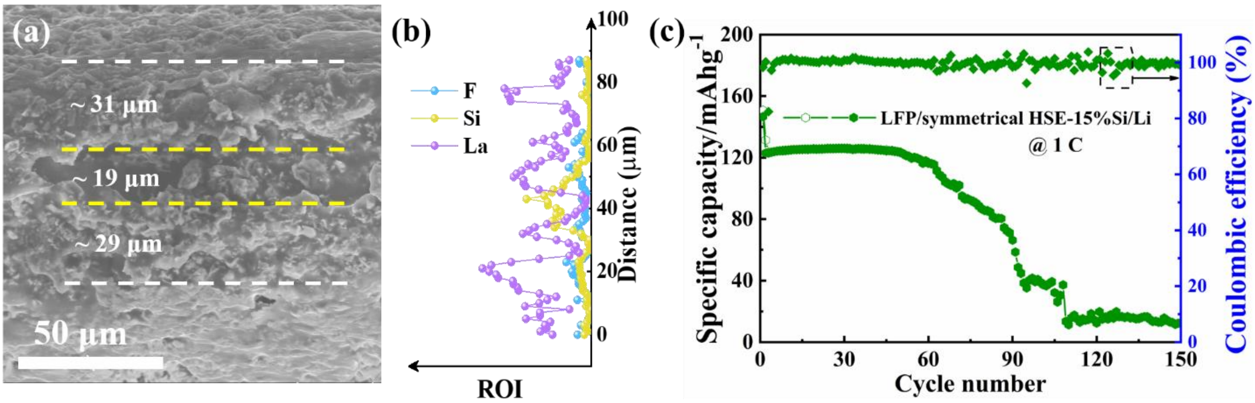

Figure S21 (a) Cross-sectional SEM image of symmetrical HSE-15%Si and (b) the EDS line scanning image. (c) Cycle performance of LFP/symmetrical HSE-15%Si/Li batteries at 1 C at 60 °C.
